# Shikonin combined with methotrexate regulate macrophage polarization to treat psoriasis

**DOI:** 10.1080/21655979.2022.2062090

**Published:** 2022-04-29

**Authors:** Tingjun Tao, Yan Chen, Bochen Lai, Jinhua Wang, Weiliang Wang, Weimian Xiao, Xushan Cha

**Affiliations:** a Department of Dermatology, The First Affiliated Hospital of Guangzhou University of Chinese Medicine, Guangzhou, Guangdong, China; b The First Clinical Academy, Guangzhou University of Chinese Medicine, Guangzhou, Guangdong, China; c Department of Dermatology, Yangjiang People’s Hospital, Yangjiang, Guangdong, China; d Key Laboratory of Dermatology, Anhui Medical University, Ministry of Education, Hefei, Anhui, China; e The Second Clinical Academy, Xinjiang Medical University, Xinshi District, Urumqi, Xinjiang Uygur Autonomous Region, China; f Department of Dermatology, Qingyuan Skin Disease Hospital, Qingyuan, Guangdong, China

**Keywords:** Shikonin, methotrexate, macrophage polarization, psoriasis

## Abstract

This study aimed to investigate whether shikonin combined with methotrexate could inhibit psoriasis progression by regulating the polarization of macrophages through *in vivo* and *in vitro* experiments. Imiquimod was administrated to the exposed skin of BALB/c mice, and shikonin and methotrexate suspension were also given by gavage. The erythema, scales and thickness were scored for mice lesions in each group, and the total score was obtained by adding the above three scores, and calculated as psoriasis area and severity index (PASI) score. The skin lesion tissue from mice was isolated and used for hematoxylin-eosin staining and immunohistochemistry assay. Drug-containing serum was prepared and administrated into mouse macrophage RAW264.7 cells, followed by simulation of LPS. The levels of tumor necrosis factor-α (TNF-α), Interleukin (IL)-1β, and IL-6 in cell supernatant were assessed using ELISA Kits and real-time PCR. In imiquimod-induced psoriasis mice, shikonin combined with methotrexate exerted protective effects by reducing erythema and PASI scores, decreasing backer score and epidermal thickness, and particularly regulating macrophage polarization. In LPS-stimulated RAW264.7 cells, shikonin combined with methotrexate regulated M1/M2 polarization and altered the levels of M1 markers. Shikonin combined with methotrexate inhibit psoriasis progression by regulating the polarization of macrophages, which may be useful in the treatment of psoriasis.

## Graphical Abstract

**Figure:**
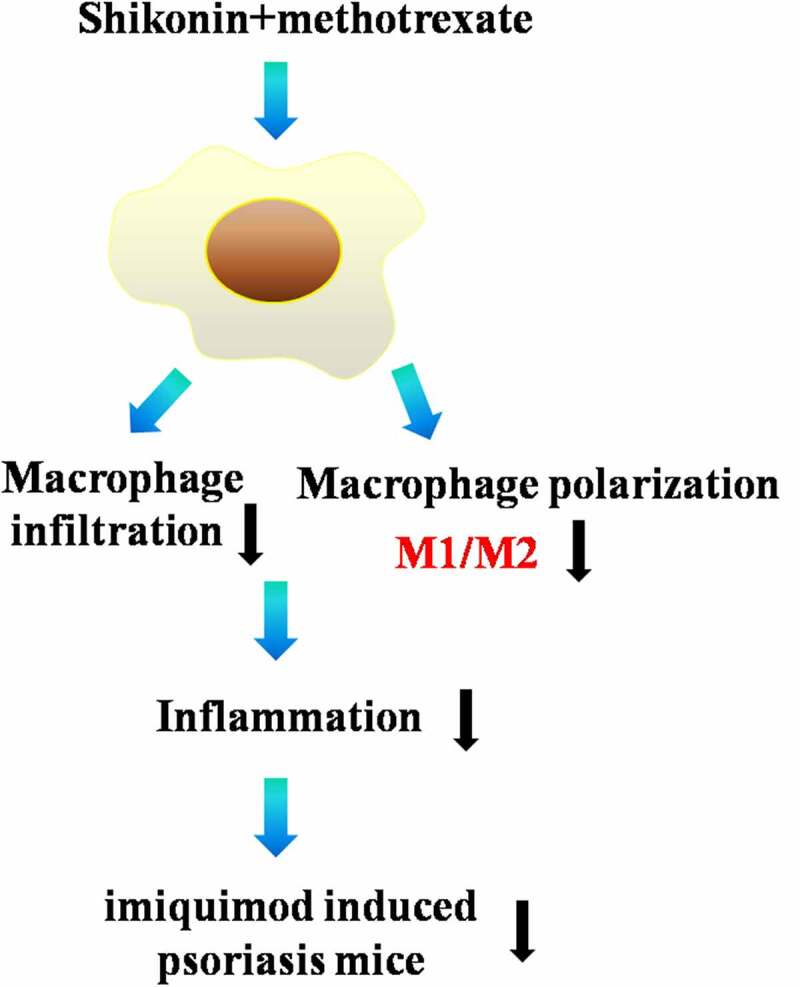

## Highlights

- In psoriasis mice, shikonin plus methotrexate exerted protective effects.
- shikonin plus methotrexate inhibit M1 type macrophage polarization in psoriasismice.
- shikonin plus methotrexate inhibit M1 type macrophage polarization in RAW264.7cells.

## Introduction

Psoriasis is a chronic inflammatory skin disease caused by abnormal immune function, and its pathological mechanisms include excessive proliferation of keratin-forming cells, neovascularization and inflammatory cell infiltration [1]. Macrophages are a key natural immune cell, and macrophage infiltration is seen at the true and epidermal junction in psoriasis patients’ lesions, suggesting that macrophages play an important role in the pathogenesis of psoriasis and influence the course of psoriasis [2].

Macrophages are heterogeneous and plastic, and can be divided into classical activated macrophages (M1 type) and alternative activated macrophages (M2 type) according to the environment, surface markers and functions of macrophages [3].M1 type macrophages can secrete a variety of inflammatory factors such as interleukin (IL)-1β, IL-6, IL-12, IL-17A and IL-23, which mainly play a pro-inflammatory role. In contrast, M2 type macrophages secrete IL-10, transforming growth factor-beta (TGF-β), C-C motif chemokine ligand (CCL) 17, CCL22, etc., which mainly have anti-inflammatory effects. A previous study reported [4] that IL-35 could reduce macrophage infiltration and the ratio of M1/M2 macrophages, suggesting that IL-35 exerts a powerful immunosuppressive effect in psoriasis. Li *et al* [5] found that the expression of M1 macrophage-related factors IL-1β, IL-6, TNF-α and inducible nitric oxide synthase (iNOS) was significantly elevated in an imiquimod-induced psoriasis mouse model. However, treatment of Chinese herbal medicine PSORI-CM02 ameliorated psoriasis progression by inhibiting M1 macrophages and promoting the expression of M2 macrophage cytokines. Therefore, by regulating the direction of M1 and M2 polarization of macrophages has an important role in the development of psoriasis.

Comfrey is one of the commonly used drugs in the treatment of psoriasis, which can be used as a single drug or in combination with other drugs, both internally and externally, with positive clinical efficacy [6]. Shikonin is the main active ingredient of comfrey, and modern pharmacological studies have concluded that shikonin has anti-inflammatory [7], anti-tumor [8], and anti-allergic [9] effects, but its mechanism of action in the treatment of psoriasis is not clear. It has been reported [10] that shikonin can inhibit the expression of iNOS and NO production in mouse macrophages RAW264.7, suggesting that shikonin can act by regulating macrophages.

Therefore, this study aimed to investigate whether shikonin combined with methotrexate could inhibit psoriasis progression by regulating the polarization of macrophages through *in vivo* and *in vitro* experiments.

## Materials and methods

### Animal and groups

A total of 50 SPF grade 6 ~ 8 weeks old BALB/c mice were purchased from Beijing Vital River Laboratory Animal Technology Co., Ltd. These mice were kept in a standard SPF laboratory room (23 ± 1°C; 12 h light/12 h dark cycle; 45–55% humidity; free access to food and water) for 2 weeks. All protocols were approved by the Ethics Committee of the Yangjiang People’s Hospital (20,220,003). Mice were randomly divided into 5 groups (n = 10 in each group): Control group, pure petroleum jelly was administrated to the exposed skin of mice once every day for 7 d. Psoriasis group, 5% imiquimod (Sichuan Mingxin Pharmaceutical Co., Ltd., H20030128) was administrated to the exposed skin of mice once every day (42 mg/day) for 7 d. Shikonin group, 5% imiquimod was administrated to the exposed skin of mice once every day (42 mg/ day) for 7 d, and 40 mg/ kg/d of shikonin suspension (sigma, S7576, purity≥98%) was also given by gavage for 7 d. Methotrexate group, 5% imiquimod was administrated to the exposed skin of mice once every day (42 mg/ day) for 7 d, and 1 mg/ kg/d of methotrexate suspension (Tonghua Maoxiang Pharmaceutical Co., Ltd., H22022674) was also given by gavage for 7 d. Shikonin + Methotrexate group, 5% imiquimod was administrated to the exposed skin of mice once every day (42 mg/ day) for 7 d, along with 40 mg/ kg/d of shikonin and 1 mg/ kg/d of methotrexate suspension were also given by gavage for 7 d. The animal model of psoriasis and drug treatment refer to the report [11], and some modifications have been made on the basis of this report.

### Measurement of PASI score

The measurement of PASI score was independently evaluated by at least two people. The PASI was scored as follows: 0 (Normal): no erythematous scales visible on the surface, lesions flush with normal skin. 1 (Mild): some of the lesions are covered with scales, mainly fine scales, lesions slightly above the normal skin surface, light red. 2 (Moderate): most of the lesions are completely or incompletely covered with scaly shoulders, scales are flaky The lesions were completely or incompletely covered with scaly shoulders, the scales were flaky, moderately elevated, the edges of the patches were round or slope shaped, red. 3 (Severe): almost all of the lesions were covered with scales, the scales were thicker in layers, the lesions were hypertrophic, the elevation was obvious, dark red. 4 (Very severe): all of the lesions. The erythema, scales and thickness were scored for mice lesions in each group, and the total score was obtained by adding the above three scores, and the trend change of skin lesion score was plotted after taking the average of daily scores of each group to observe the changes of mouse lesions.

### Hematoxylin-eosin staining

The skin lesion tissue from mice was isolated, fixed and then embedded by 4% paraformaldehyde and paraffin, respectively. Then, the embedded tissues were cut into 5 µm sections, and stained with hematoxylin for 5 min and eosin for 1 min. The stained sections were evaluated under a light microscope. The epidermal thickness was measured and Baker scores were obtained as follows: Munro small abscesses were found in the stratum corneum, score 2.0, hyperkeratosis, score 0.5, and incomplete keratinization, score 1.0. Thinning or loss of granular layer in the epidermis, score 1.0, thickening of spine layer, score 1.0. The prolongation and undulation of dermal protrusions, score 0.5, 1.0 and 1.5 according to mild, moderate and severity, respectively. The infiltration of single or multinucleated cells in the dermis, score 0.5, 1.0 and 1.5 according to mild, moderate and severity, respectively. Supratentorial papillae, score 0.5, and capillary dilatation, score 0.5. The measurement of hematoxylin eosin staining score was independently evaluated by at least two people.

### Immunohistochemistry

Sections (4 μm) of skin lesion tissue from mice were mounted on glass slides, stained with 3,3-diaminobenzidine tetrahydrochloride (Dojindo, Kumamoto, Japan), and counterstained with hematoxylin and processed for immunohistochemical detection of F4/80 by mouse monoclonal F4/80 antibody (Serotec, MCA 497 R, Raleigh, NC) according to standard immunoperoxidase procedure.

### Preparation of drug-containing serum

Mice were anesthetized by intraperitoneal injection of chloral hydrate (7%; 0.5 mL/100 g). The abdominal cavity of the mice was dissected, and blood samples were drawn from the abdominal aorta and left to stand for 2 hours. To induce blood clotting, blood samples were stored in a refrigerator (4°C; 4 hours) and then centrifuged (3000 r/min; 15 min). The supernatant obtained was inactivated (56°C; 30 min) and sterilized (millimolar filters of 0.2 μm). Finally, the sera were stored at −70°C for the following analysis.

### Cell culture and groups

RAW 264.7 cell line was purchased from the American Type Culture Collection (ATCC). Cells were cultured in RPMI 1640, with supplementary of fetal bovine serum (FBS; 10%), penicillin (100 IU/ml) and streptomycin (100 μg/mL), and maintained (37°C; 5% CO_2_). RAW264.7 cell suspension, adjusted cell density and inoculated in 6-well plates for 12 hours. Cells were randomly divided into 5 groups: Control group, cells were treated with sera (15%) from control mice for 12 hours. LPS group, cells were treated with sera (15%) from control mice for 12 hours, followed by stimulated with 100 ng/ml of LPS for 12 hours. Shikonin group, cells were treated with sera (15%) from mice in Shikonin group for 12 hours, followed by stimulated with 100 ng/ml of LPS for 12 hours. Methotrexate group, cells were treated with sera (15%) from mice in Methotrexate group for 12 hours, followed by stimulated with 100 ng/ml of LPS for 12 hours. Shikonin +Methotrexate group, cells were treated with sera (15%) from mice in Shikonin +Methotrexate group for 12 hours, followed by stimulated with 100 ng/ml of LPS for 12 hours.

### Flow cytometry

Mouse macrophage RAW264.7 cells were seeded in triplicate onto 6-well (1 × 10^5^ cells/well). Cells were blocked using the Ultra V blocker (Thermo Fisher Scientific, San Jose, CA, USA), and incubated with APC anti-CD86, or PE anti-CD206 antibody (1:50). Finally, cells were fixed and measured using a Guava easy Cyte 8 Millipore flow cytometer.

### ELISA

The levels of tumor necrosis factor-α (TNF-α), IL-1β, IL-6 in cell supernatant were assessed using mice TNF-α ELISA Kit, IL-1β ELISA Kit, IL-6 ELISA Kit (Elabscience, Wuhan, China) as per the manufacturers’ instruction. The optical density values of the samples were measured using an enzyme marker (Thermo Fisher Scientific, San Jose, CA, USA).

### Real-time PCR

The total RNA was extracted with TRIzol (Invitrogen, Carlsbad, CA, United States) and converted into first-strand cDNA using PrimeScript^TM^ RT reagent Kit (TaKaRa Biotechnology, Co., Ltd., Dalian, China). Quantitative real-time PCR (qRT-PCR) experiments were performed with SYBR Green I in a Light Cycler 96 (Roche Applied Science, Mannheim, Germany). The primers were as follows: iNOS, 5'-CCCTTCCGAAGTTTCTGGCAGCAGC-3' and 5' -GGCTGTCAGAGCCTCGTGGCTTTGG-3'; IL-1β, 5'-AAACAGATGAAGTGCTCCTTCCAGG-3' and 5' -TGGAGAACACCACTTGTTG CTCCA';TNF-α, 5'-TTGACCTCAGCGCTGAGTTG-3' and 5' -CCTGTAGCCCACGTCGTAGC-3';

GAPDH, 5'-GCACCACCAACTGCTTAGCA-3' and 5'-GTCTTCTGGGTGGCAGTGATG-3'. For normalization, GAPDH gene was used as a reference gene and calculated by the 2^−ΔΔCt^ method.

### Statistics

GraphPad 6.0 software was used for statistical analysis. Data were represented as mean ± SEM. Anderson-Darling test was used to test the data normality distribution. The statistical difference between two groups was compared using unpaired student’s t tests. Comparisons among multiple groups were analyzed using one-way analysis of variance (ANOVA) with Tukey’s post hoc test. *p* < 0.05 was considered as significant.

## Results

### Shikonin combined with methotrexate reduced erythema and PASI scores in imiquimod-induced psoriasis mice

**Figure 1.**
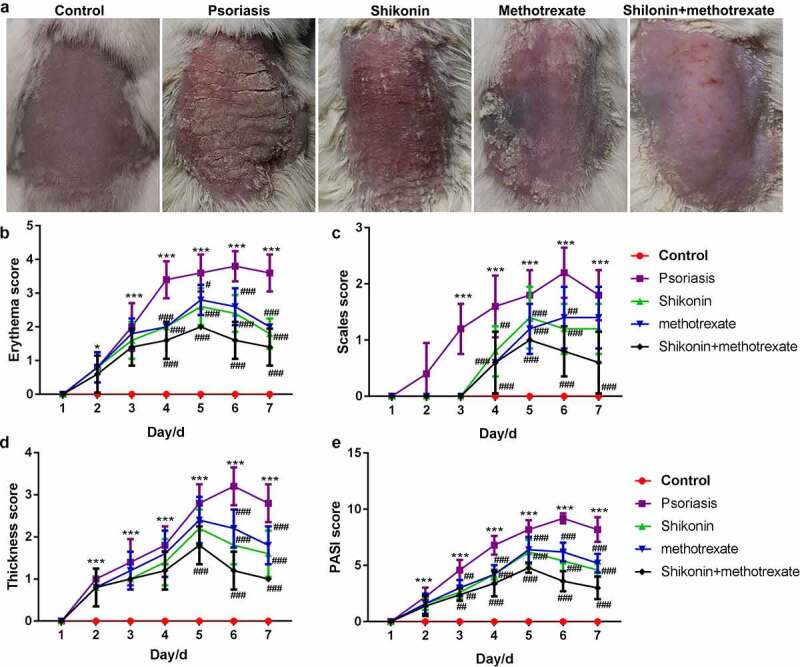
Shikonin combined with methotrexate reduced erythema and PASI scores in imiquimod-induced psoriasis mice. BALB/c male mice of SPF grade (6–8 weeks old) were coated with 5% imiquimod for 7 d, followed by administration with shikonin and methotrexate by gavage for 7 d, and finally optically photographed (a). The erythema score (b), scales score (c), thickness score (d) and PASI score (e) in mice in Control, psoriasis, shikonin, methotrexate and shikonin + methotrexate groups. ***p < 0.001 *vs*. Control group. ^#^*p* < 0.05, ^##^*p* < 0.01 and ^###^*p* < 0.001 *vs* .psoriasis group.

To gain a clear understanding on the functional effects of shikonin combined with methotrexate in the treatment of psoriasis, BALB/c male mice of SPF grade (6–8 weeks old) were coated with 5% imiquimod for 7 d, followed by administration with shikonin and methotrexate by gavage for 7 d, and finally optically photographed (Figure 1a). Despite those in the control group, the erythema score, scales score, thickness score and PASI score in shikonin + methotrexate were the lowest, while were highest in imiquimod-induced psoriasis mice. Taken together, shikonin combined with methotrexate could exert protective effects on imiquimod-induced psoriasis mice by reducing erythema, psoriasis area and severity.

### Shikonin combined with methotrexate reduced backer score and epidermal thickness in imiquimod-induced psoriasis mice

**Figure 2.**
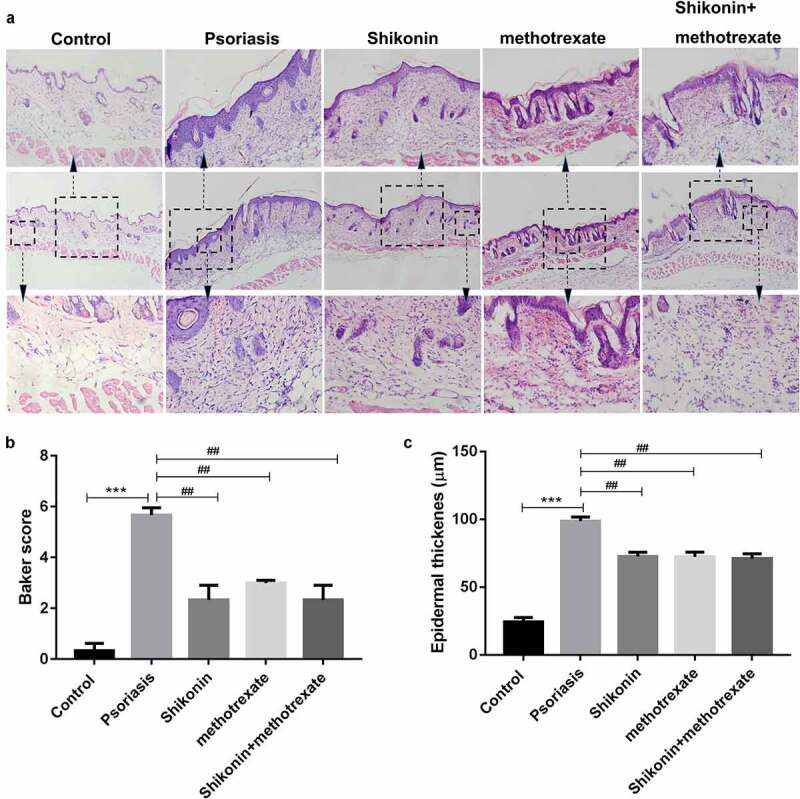
Shikonin combined with methotrexate reduced backer score and epidermal thickness in imiquimod-induced psoriasis mice. The skin lesion tissue from mice in Control, psoriasis, shikonin, methotrexate and shikonin + methotrexate groups was isolated, stained by hematoxylin-eosin and optically photographed (a). The statistical data showed the backer score (b) and epidermal thickness (c) from (A). ***p < 0.001 *vs*. Control group. ^##^*p* < 0.01 *vs*. psoriasis group.

To evaluate the skin damaged tissues of mice, the skin damaged tissues of mice were isolated and stained with hematoxylin eosin, and then photographed optically. As shown in Figure 2a, we present three groups of hematoxylin eosin staining pictures with different magnification. The statistical data showed that the backer score and epidermal thickness in imiquimod-induced psoriasis mice were higher than that in Control mice, which were respectively decreased by shikonin and methotrexate treatment (Figure 2b-2c). Thus, shikonin combined with methotrexate could exert protective effects on imiquimod-induced psoriasis mice by reducing erythema, psoriasis area and severity.

### Shikonin combined with methotrexate regulated macrophage polarization in imiquimod-induced psoriasis mice

**Figure 3.**
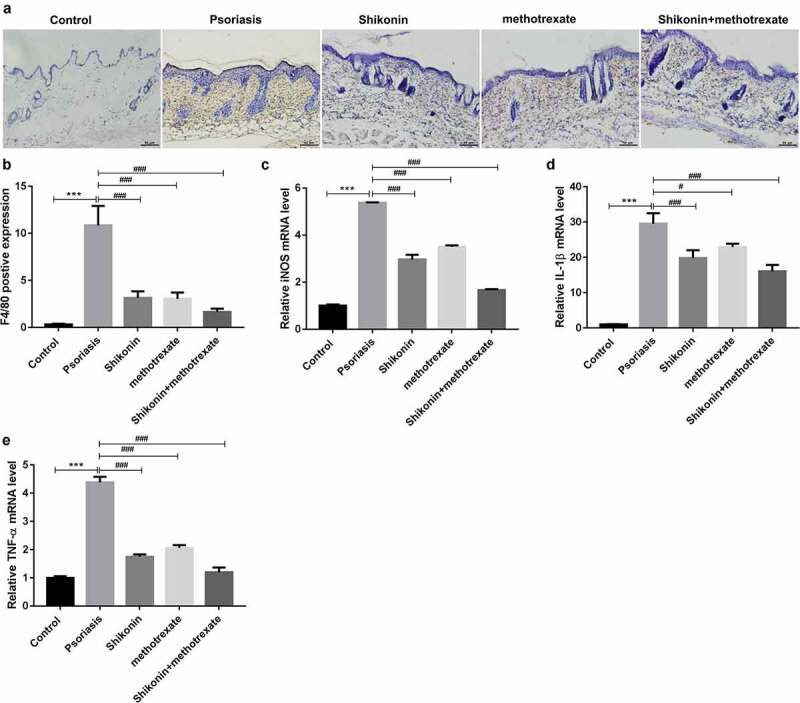
Shikonin combined with methotrexate regulated macrophage polarization in imiquimod-induced psoriasis mice. Macrophage infiltration in skin lesion tissue from mice in Control, psoriasis, shikonin, methotrexate and shikonin + methotrexate groups was observed under immunohistochemistry via staining by F4/80, and optically photographed (a). The statistical data showed the F4/80 positive expression (b) from (A). The mRNA expressions of macrophage M1-type markers iNOS (c), IL-1β (d), TNF-α (e) in skin lesion tissue from mice in Control, psoriasis, shikonin, methotrexate and shikonin + methotrexate groups. Scar bar = 50 µm, ***p < 0.001 *vs*. Control group. ^#^*p* < 0.05 and ^###^*p* < 0.001 *vs*. psoriasis group.

To identify the infiltration and polarization of macrophages, we detected the expression of F4/80 by immunohistochemistry and the expression of iNOS, IL-1β and TNF-α by RT-PCR. The statistical data showed that the F4/80 positive expression in imiquimod-induced psoriasis mice were higher than that in Control mice, which were decreased by shikonin and methotrexate treatment (Figure 3a,b). Besides, the mRNA expressions of macrophage M1-type markers iNOS, IL-1β, TNF-αin imiquimod-induced psoriasis mice were higher than that in Control mice, which were decreased by shikonin and methotrexate treatment (Figure 3c-3e). Totally, shikonin combined with methotrexate could exert protective effects on imiquimod-induced psoriasis mice by inhibiting M1 type macrophage polarization.

### Shikonin combined with methotrexate regulated macrophage polarization in LPS-stimulated mouse macrophage RAW264.7 cells

**Figure 4.**
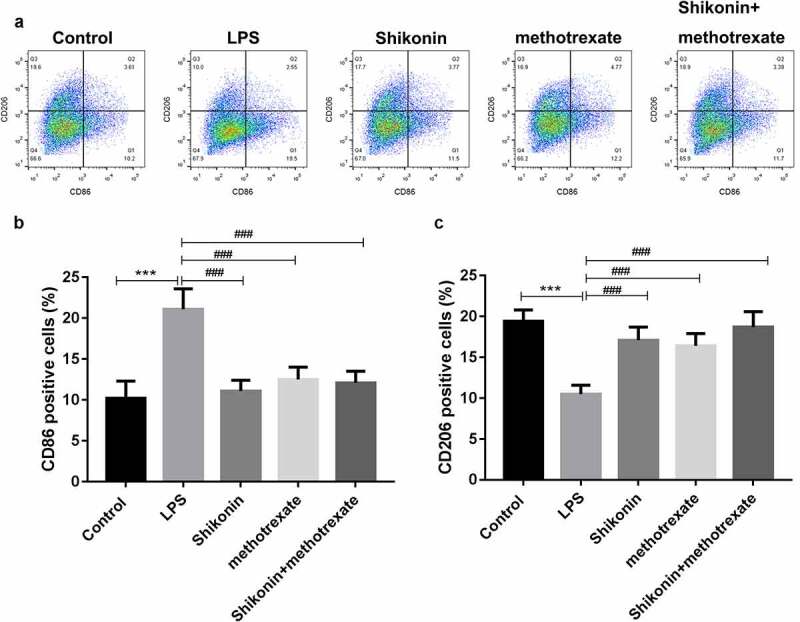
Shikonin combined with methotrexate regulated M1/M2 polarization in LPS-stimulated mouse macrophage RAW264.7 cells. Mouse macrophage RAW264.7 cells were stimulated with LPS, followed by shikonin and methotrexate treatment, and flow cytometry was used to detect the ratio of M1 and M2 macrophage by determining the expression levels of M1 and M2 cell markers, CD86 and CD806 (a). The statistical data showed the number of CD86(b) and CD206(c) in macrophage RAW264.7 cells in Control, LPS, shikonin, methotrexate and shikonin + methotrexate groups. ***p < 0.001 *vs*. Control group. ^###^*p* < 0.001 *vs*. LPS group.

To explore the effect of shikonin and methotrexate on macrophage polarization in vitro, mouse macrophage RAW264 was stimulated with LPS, and then treated with shikonin and methotrexate. The proportion of M1 and M2 macrophages was measured by flow cytometry (Figure 4a). The statistical data showed that the number of CD86 was significantly increased in the LPS group, while was decreased by shikonin and methotrexate treatment. Besides, the number of CD206 was significantly decreased in the LPS group, while was increased by shikonin and methotrexate treatment.

### Shikonin combined with methotrexate regulated the levels of M1 markers in LPS-stimulated macrophage RAW264.7 cells

**Figure 5.**
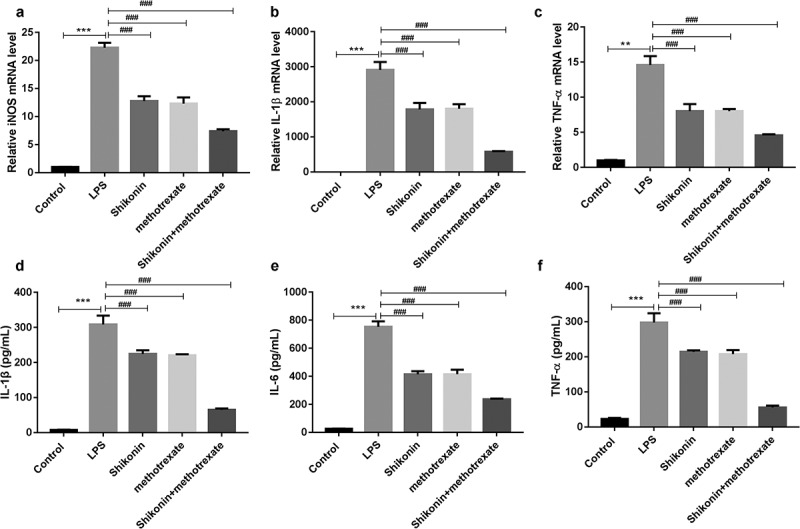
Shikonin combined with methotrexate regulated the levels of M1 and M2 markers in LPS-stimulated mouse macrophage RAW264.7 cells. The expression levels of M1 marker iNOS (a), IL-1β (b), TNF-α (c) in macrophage RAW264.7 cells in Control, LPS, shikonin, methotrexate and shikonin + methotrexate groups were measured by qRT-PCR. ELISA assay was used to detect IL-1β (d), IL-6 (e), TNF-α (f) levels in the culture supernatant of macrophage RAW264.7 cells in Control, LPS, shikonin, methotrexate and shikonin + methotrexate groups. **p < 0.001 and ***p < 0.001 *vs*. Control group. ^###^*p* < 0.001 *vs*. LPS group.

To further characterize the effect of shikonin and methotrexate on macrophage polarization in vitro, the expression levels of M1 marker iNOS, IL-1β, TNF-α were measured by qRT-PCR, and ELISA assay was used to detect TNF-α, IL-1β and IL-6 levels in the culture supernatant. The results, as shown in Figure 5, indicated that LPS treatment significantly increased the mRNA expressions of iNOS, IL-1β and TNF-α. However, shikonin and methotrexate treatment decreased the expressions of iNOS, IL-1β and TNF-α. Consistently, ELISA assay showed that LPS treatment significantly increased the levels of TNF-α, IL-1β and IL-6. However, shikonin and methotrexate treatment increased the levels of TNF-α, IL-1β and IL-6. These data suggested that shikonin combined with methotrexate inhibits M1 type macrophage polarization in LPS stimulated RAW264.7 cells

## Discussion

Psoriasis is a complex disease involving endothelial cells, keratinocytes, and immune cells, such as T lymphocytes and macrophages [12–15]. It has been reported that topical application of imiquimod resulted in time-dependent thickening, scaling, erythema and inflammation of the affected skin [16], which were similar in our investigation. However, in imiquimod-induced psoriasis mice, shikonin combined with methotrexate exerted protective effects by reducing erythema and PASI scores, decreasing backer score and epidermal thickness, indicating its protective role.

The first study of the efficacy of methotrexate in the treatment of psoriasis was published in 1958 [17], and the first guide lines on its use in dermatology appeared in 1972 [18]. Shikonin is a 288-kDa liposoluble naphthoquinone derived from *Lithospermum erythrorhizon*, which could suppress IL-17-induced production of cytokines associated with psoriasis by inhibiting the JAK/STAT3 signaling pathway [19] and suppression of CEBPD downregulation [16]. Another *in vivo* experiment also indicated that intragastrically administered shikonin effectively improved lesions in imiquimod-induced mice with psoriasis and increased the number of iTreg cells in the spleen and their secretion [20]. Besides, Shikonin could inhibit IL-17-induced proliferation in HaCaT cells and secretion of relevant cytokines and recruit leukocytes by inhibition of chemokines to exert protective role in psoriasis treatment [21].

In particular, shikonin combined with methotrexate regulated macrophage polarization, evidenced by the decreased number of CD86, increased number of CD206, and altered levels of M1-type markers iNOS, IL-1β, TNF-α and macrophage M2-type markers Arg-1, IL-10. In LPS-stimulated mouse macrophage RAW264.7 cells, shikonin combined with methotrexate also regulated M1/M2 polarization and altered the levels of M1 and M2 markers, which were consistent with the *in vivo* experiments. It has been reported that IL-23 induced macrophage polarization and IL-23-treated macrophages significantly promote the dermatitis pathogenesis in a psoriasis-like mouse model [22]. Li *et al*. indicated that PSORI-CM02, a novel Chinese medicine, may possess therapeutic action in psoriasis treatment by regulating the infiltration and polarization of macrophages in the dermal layer [5]. Miki *et al*. indicated that 4–1BBL signaling regulates macrophage polarization and contributes to imiquimod-induced psoriasis by sustaining inflammation [23]. Taken together, these investigations indicated that shikonin combined with methotrexate on regulating M1/M2 polarization, and thus exerts a protective effect against psoriasis. More mechanism involved need deeper investigations.

## Conclusion

Overall, this study indicated that shikonin combined with methotrexate inhibit psoriasis progression by regulating the polarization of macrophages, which may be useful in the treatment of psoriasis.
